# Does attendance at anatomy practical classes correlate with assessment outcome? A retrospective study of a large cohort of undergraduate anatomy students

**DOI:** 10.1186/s12909-015-0515-y

**Published:** 2015-12-23

**Authors:** David G Gonsalvez, Matthew Ovens, Jason Ivanusic

**Affiliations:** 1 Department of Anatomy and Neuroscience, University of Melbourne, Melbourne, VIC 3010 Australia; 2 YourStatsGuru, Melbourne, Australia

**Keywords:** Dissection, Prosection, Anatomy, Education, Assessment

## Abstract

**Background:**

Anatomy in medical curricula is typically taught via pedagogy consisting of didactic lectures combined with a practical component. The practical component often includes traditional cadaveric dissection classes and/or workshops utilizing anatomical models, carefully prosected cadaveric material and radiology. The primary aim of this study was to determine if there is an association between attendance at practical classes in anatomy and student assessment outcomes. A secondary aim was to determine if student assessment outcomes were better when students preferentially attended workshops or prosection style practical classes.

**Method:**

We retrospectively examined practical attendance records and assessment outcomes from a single large anatomy subject (approx. 450 students) to identify how attendance at anatomy practical classes correlates with assessment outcome.

**Results:**

Students who scored above the median mark for each assessment attended significantly more practical classes than students who scored below the median assessment mark (Mann Whitney; *p* < 0.001), and students who attended more than half the practical classes had significantly higher scores on assessments than students that attended less than half the practical classes (Mann Whitney; *P* < 0.01). There was a statistically significant positive correlation between attendance at practical classes and outcomes for each assessment (Spearman’s correlation; *p* < 0.01). There was no difference in assessment outcomes for students who preferentially attended more dissection compared to prosection style classes and vice versa (Mann Whitney; *p* > 0.05).

**Conclusions:**

Our findings show there is an association between student attendance at practical classes and performance on anatomy assessment.

## Background

Anatomy in medical curricula is typically taught via pedagogy consisting of didactic lectures combined with a practical component. Since the 16^th^ century the practical component has predominantly focused around human cadaveric dissection [1]. In recent times, restrictions on time and resource have resulted in institutional specific modifications to this traditional pedagogy. In particular, the practical component has undergone rapid reform. In many cases, cadaveric dissection has been replaced by workshops that use combinations of anatomical models, carefully prosected cadaveric material and radiology. However, only limited objective experimental evidence exists for how these different anatomy practical teaching methods impact on the student experience and functional understanding of anatomy [2]. Because of the explicit objectives in most of these experimental studies, attendance at either dissection or prosection style practical classes is mandatory, and students that do not attend all classes are excluded from the data analyzed. Thus it is not clear if varying levels of attendance at classical dissections and/or workshops based on prosected material can have an impact on student outcomes. This important caveat is often overlooked in studies of teaching methods, and is certainly the case for studies related to anatomy practical classes, for which we could find no literature that explored the relationship between attendance and student outcome.

The primary aim of this study was to determine if there is an association between attendance at practical classes in anatomy and student assessment outcomes. We retrospectively examined attendance records and normal assessment outcomes from a single, large cohort of students (approximately 450) studying anatomy at the University of Melbourne to identify how attendance at different kinds of anatomy practical classes correlates with assessment outcomes. A secondary aim was to determine if assessment outcomes were better when students preferentially attended workshops or dissection style practical classes.

## Methods

### Subject and student description

The study was approved by the University of Melbourne Human Research Ethics committee. Data acquired for analyses were collected routinely as part of our normal assessment procedures, to refine our teaching practice and to provide students with feedback. All data were de-identified by student administrators prior to analysis. We retrospectively explored the relationship between attendance and assessment outcomes for students enrolled in an anatomy subject (ANAT30008) at the University of Melbourne. This subject is taught to approximately 450 undergraduate students studying in the third year of their degree. The anatomical regions covered in the subject are Thorax, Abdomen, Pelvis, and Ear, Nose and Throat. During the semester, the students receive 36 h of didactic lectures and 30 h of practical classes. The lectures are all recorded so that they can be re-accessed or downloaded by students using an online learning management system at a time that they are preparing for exams or if they were unable to attend the lecture in person. Whilst we are unable to derive data about online access for individual students, we do note that the number of times each lecture was accessed far exceeded the number of students, suggesting that most, if not all, students accessed/downloaded each lecture at least once. All students have a similar background and have completed subjects containing the same pre-requisite material.

### Practical teaching program

Throughout the semester students have the option to attend a total of ten practical classes (with each class running for three hours). There are five practical classes dedicated to classical dissection and five dedicated to a workshop style that predominantly utilizes prosections for most of the teaching.

#### Classical dissection classes

The dissection classes follow a classical dissection style where a maximum of 8 students are assigned to each cadaver. There is one demonstrator supervising the dissection for two cadavers (16 students maximum). The students are allowed to self-enroll into dissection groups with their study partners to facilitate their learning. They follow the dissection process laid out in the An@tomedia ‘Practical Anatomy Guide and Dissector’ [3]. All demonstrators have a minimum qualification of a medical degree, or a PhD in a discipline related to anatomy with significant experience in anatomy demonstration. The demonstrators are not involved in the examination process and have no knowledge of the assessments, so they are unable to ‘telegraph’ to the students what is on the assessments.

#### Workshop practical classes

In workshop practical classes, a large range of prosected specimens, plastinated specimens, bones and plastic models are made available to students. Prior to the workshops, students receive notes (learning tasks) that highlight the material available for them in the class. The students can make their own decisions on how much time to spend exploring different kinds of material. Students are not assigned to any particular group and there are no formal stations through which they rotate. The ratio of demonstrators to the number of students is identical to that for the dissections (1 demonstrator to 16 students). Qualifications of demonstrators for these classes were the same as for dissection classes. The demonstrators are instructed not to deliver didactic tutorials, but rather to move around the room and help students who need assistance with the material. Thus the learning process in workshop classes is self-directed.

### Collection of attendance records

The demonstrators took attendance records close to the end of each practical class to ensure that students did not come to class, mark their name off and leave without participating sufficiently. Although we strongly recommended that students attend all classes, the choice to attend was up to the student and missing class had no consequence, other than missing out on the material available in that particular class. This created a situation in which we had graded levels of attendance at either workshop and/or dissection classes, and allowed us to explore correlations between varying attendance at practical classes and assessment outcomes.

### Assessment styles

**Fig. 1:**
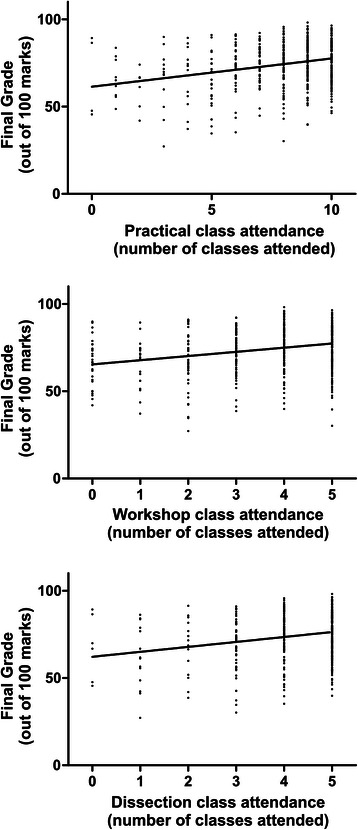
Scatterplots of attendance at all practical classes, workshop style classes and dissection classes vs assessment outcome (final grade). A linear best fit model was used to define a regression line for each graph

The subject involved four distinct assessments that make up a final grade (two ongoing mid-semester tests and two end-of-semester exams). The ongoing tests were made up of 30 multiple choice questions each, contributing to a total of 20 % of the final grade for each student. These ongoing tests were not included in the analysis of individual assessment outcomes in the present study because they were small summative assessments that we did not believe to be a good indicator of overall success when explored in isolation. However, they did contribute to the final grade for the students. The end-of-semester exams consisted of one theory and one practical exam. The theory exam contained 30 multiple choice questions and six, two page written questions, together contributing to 50 % of the final grade for each student. The written component of this assessment allowed students to communicate the extent of their knowledge and was assessed with marking criteria that rewarded students who demonstrated a higher level of anatomical knowledge (and application of that knowledge). The practical exam contained 12 images of prosected specimens, each image associated with 4 multiple choice questions relating to the material, and another 30 multiple choice question relating directly to dissection, together contributing to 30 % of the final grade. The 12 images were of specimens from our collection, which were freely available for exploration in the prosection style classes, but were also available in An@tomedia (online student resource) and were typical of the sorts of images in students’ recommended texts (including anatomical photographic atlases). Thus there was ample opportunity for students to engage with these sorts of images even if they did not attend the practical classes. The MCQs relating directly to dissection were carefully constructed to test whether students could make predictions about the dissection process based on their level of understanding of anatomy, regardless of whether or not they had attended the dissection program. However, students who engaged in the dissection program were expected to perform better on these latter questions because we assume they had more practice in making the sorts of predictions required to answer the questions. Interestingly, our data show that that many students that did not attend practical classes at all, or only attended a few classes, were able to perform well on these assessments (Fig. 1). This reinforces the notion that questions were constructed in a way that essential information provided in practical classes was available from alternative resources.

### Data collection

Attendance records were collected by the demonstrators as described above, and were compiled by our student administrators at the end of semester into an excel file that also contained student assessment outcomes for the entire subject. The assessment outcomes were obtained after our board of examiners had ratified the results and therefore consisted of a final outcome for each student. Students who had not completed the same pre-requisite subjects required for entry into this subject were excluded from this study, as were students that did not complete all assessment tasks in the subject. After exclusions, data from 446 students were analyzed.

### Statistical testing

For statistical analysis we used either SPSS or Graph Pad Prism (version 5.0a). We reported descriptive statistics (parametric and non-parametric) for attendance and assessment. We used a Mann Whitney *U*-test (MW) to determine if there was a difference in attendance for students who scored above the median for each assessment outcome compared to students who scored below the median, and also to test whether there was a difference in assessment outcome for students who attend less than half of the classes compared to those who attended more than half of the classes. This test is a non-parametric alternative to the *t* test for comparing data from two independent groups [4]. The median score for the first of these analyses was chosen arbitrarily on the basis that we believe this to be a good indicator of what students feel is an appropriate indicator of success, and provided us with balanced group sizes (number of students) for analysis. We used Spearman’s correlation to determine the degree of association between attendance and assessment outcome for all students. A Spearman’s correlation coefficient (r_s_) that is positive indicates that two variables are increasing with each other [4]. If the magnitude of the change in ranks is the same for each variable then r_s_ should = 1, but an r_s_ close to 1 is not necessary to indicate association. Considerably lower values for r_s_ still indicate an association between two variables. Spearman’s analysis can be performed with a simple test of significance of the null hypothesis (that there is no association), and this provides a more robust indicator of whether or not an association is significant. Indeed in our present study we predicted that r_s_ would be positive, but not get close to 1, as we did not expect students assessment marks to increase in increments that followed the magnitude of increments possible in attendance. Therefore, we report the results of hypothesis testing with the Spearman’s analysis to indicate whether or not the associations are indeed significant. We used a MW test to determine if there was a difference in scores for students who preferentially attended workshops (more workshops than dissections) and those who preferentially attended dissections (more dissections than workshops). For each of the above analyses, testing was performed for each of the different types of attendance (workshops and dissection classes, and total practical classes) and each of the different assessment outcomes (final grade, theory exam grade and practical exam grade). An alpha level of 5 % was used for all statistical significance testing performed (*P* < 0.05 denotes statistical significance).

### Ethics clearance

This study was approved by the University of Melbourne Human Ethics Research Committee.

## Results

### Descriptive statistics

Results from all 453 students enrolled in the subject at the end of semester were included in this study. Students who had withdrawn prior to the end of semester were not considered. Only a small proportion of these enrolled students had not completed the same pre-requisite study or had not completed some of the assessments. After exclusions, data from 446 students were analyzed.

#### Attendance

The raw data for attendance at practical classes are summarized in Table 1. Overall, the attendance for students was high. However, there were clearly many students who did not attend many of the classes. Only 30 % of students attended all classes and some students did not attend at all (Table 1). Approximately 55 % of students attended all of the dissections, but a lesser proportion of students attended all workshops (Table 1). Attendance at dissection classes was significantly higher than attendance at workshops (MW; *P* < 0.001).

**Table 1:** The distribution of students across different levels of attendance

Total number of practicals attended	Number of students (%)
0	4 (1)
1	9 (2)
2	6 (1)
3	11 (2)
4	13 (3)
5	23 (5)
6	35 (8)
7	41 (9)
8	70 (16)
9	102 (23)
10	132 (30)
Number of workshops attended	Number of students (%)
0	27 (6)
1	19 (4)
2	44 (10)
3	74 (17)
4	108 (24)
5	174 (39)
Number of dissections attended	Number of students (%)
0	6 (1)
1	15 (3)
2	14 (3)
3	46 (10)
4	121 (27)
5	244 (55)

#### Assessment outcomes

There was a broad range of grades for each assessment. The mean (± SEM) final grade for students was 74.1 ± 0.6 % (range: 27.1–98.2). Students generally performed better on the practical exam (mean ± SEM 77.5 ± 0.6 %; range 32.5–98.7) than the theory exam (mean ± SEM 70.5 ± 0.7 %; range: 17.8–98.3). This was also reflected in the proportion of students that failed each assessment (Theory exam 11 %; Practical exam 3.4 %).

### Statistical analyses

#### Is there a difference in attendance for students who score above the median assessment score compared to students who scored below the median?

For each assessment outcome, we compared the attendance behaviors of students who scored above the median grade with students who scored below the median grade (Table 2). In almost all cases tested, students who scored above the median grade for each assessment outcome attended significantly more practical classes compared to students who scored below the median grade (MW; *P* < 0.01; Table 2).

**Table 2:** Man Whitney U-tests comparing attendance for students who scored above the median assessment mark with attendance for students who scored below the median assessment mark

Assessment	Attendance type	Above or below median mark	Mean (SEM)	25 % Percentile	75 % Percentile	Median	P value	Significant *P* < 0.01**
Final Grade	All Practicals	Below Median	7.4 (0.2)	6.0	9.0	8.0	<0.0001	**
		Above Median	8.4 (0.1)	8.0	10.0	9.0		
	Workshop	Below Median	3.3 (0.1)	2.0	5.0	4.0	<0.0001	**
		Above Median	4.0 (0.1)	3.0	5.0	4.0		
	Dissection	Below Median	4.1 (0.1)	4.0	5.0	4.5	0.0087	**
		Above Median	4.4 (0.1)	4.0	5.0	5.0		
Theory Grade	All Practicals	Below Median	7.4 (0.2)	6.0	10.0	8.0	0.0002	**
		Above Median	8.4 (0.1)	7.0	10.0	9.0		
	Workshop	Below Median	3.3 (0.1)	2.0	5.0	4.0	<0.0001	**
		Above Median	4.0 (0.1)	3.0	5.0	4.0		
	Dissection	Below Median	4.1 (0.1)	4.0	5.0	5.0	0.056	No
		Above Median	4.4 (0.1)	4.0	5.0	5.0		
Practical Grade	All Practicals	Below Median	7.4 (0.2)	6.0	9.0	8.0	<0.0001	**
		Above Median	8.3 (0.1)	7.0	10.0	9.0		
	Workshop	Below Median	3.4 (0.1)	2.0	5.0	4.0	0.0002	**
		Above Median	3.9 (0.1)	3.0	5.0	4.0		
	Dissection	Below Median	4.0 (0.1)	3.8	5.0	4.0	0.0018	**
		Above Median	4.4 (0.1)	4.0	4.0	4.0		

#### Is there a difference in assessment outcome for students who attended more than half of the practical classes compared to students who attended less than half the practical classes?

We compared assessment outcomes for students who attended more than half of the practical classes with students who attended less than half the practical classes (Table 3). Comparisons were made for each type of practical class (workshop and dissection) and also for all practical classes combined. In all cases tested, students who attended more than half the practical classes had significantly higher scores on assessments than students attended less than half the practical classes (MW; *P* < 0.01; Table 3).

**Table 3:** Man Whitney U-tests comparing scores for students who attended more than half the practicals (high attendance) with students who attended less than half the practicals (low attendance)

Assessment	Attendance type	Attendance	Mean (SEM)	25 % Percentile	75 % Percentile	Median	P value	Significant *P* < 0.01**
Final Grade	All Practicals	High	75.7 (0.6211)	67.82	84.81	77.86	<0.0001	**
		Low	65.43 (2.381)	52.4	76.83	67.42		
	Workshop	High	76.6 (0.7199)	68.82	85.25	78.74	<0.0001	**
		Low	67.18 (1.607)	56.48	77.2	68.6		
	Dissection	High	75.65 (0.6294)	67.84	84.81	77.67	0.0006	**
		Low	65.51 (2.8)	50.54	80.92	66.8		
Theory Grade	All Practicals	High	36.29 (0.3582)	32.17	41.42	37.45	<0.0001	**
		Low	30.06 (1.443)	21.64	36.74	30.23		
	Workshop	High	36.72 (0.4181)	32.62	41.74	37.65	<0.0001	**
		Low	31.1 (0.9361)	25.5	37.64	31.57		
	Dissection	High	36.18 (0.3639)	31.87	41.43	37.28	0.0011	**
		Low	30.25 (1.727)	21.63	39.82	30.23		
Practical Grade	All Practicals	High	23.62 (0.1763)	21.43	26.1	24.16	0.0008	**
		Low	21.55 (0.6015)	18.7	24.75	22.6		
	Workshop	High	23.9 (0.1989)	21.63	26.3	24.55	<0.0001	**
		Low	21.87 (0.4274)	19.09	24.94	22.21		
	Dissection	High	23.64 (0.178)	21.43	26.1	24.16	0.0056	**
		Low	21.42 (0.7752)	18.41	25.23	22.8		

#### Correlation between attendance at practical classes and assessment outcomes

In all cases, a positive association between practical attendance and assessment outcomes was observed (Spearman’s correlation analysis; r_s_ range 0.147–0.234; eg. Fig. 1). Whilst the r_s_ was relatively small, it is important to note that it does indicate a clear association between attendance and each assessment outcome, and also that we did not predict r_s_ would be close to 1. In addition, hypothesis testing revealed that these associations were always statistically significant (*P* < 0.005).

#### Does preferential workshop attendance lead to better outcomes than preferential dissection attendance?

We explored the possibility that preferential attendance at either workshop or dissection classes resulted in better assessment outcomes. There was no difference in assessment outcome for students who attended more workshops than dissections compared to students who attended more dissections than workshops (MW; *P* > 0.05). This was true for each of the assessment outcomes studied.

## Discussion

We present a number of key findings in the present study. 1) Students who score above the median assessment scores attend significantly more practical classes than students who score below the median assessment scores, and this is also true for each of the practical class types. 2) Students who attend more than half of their practical classes score better on assessments than students who attend less than half their practical classes. 3) There is a positive correlation between attendance at practical classes and assessment outcomes, and this is also true for each of the practical class types. 4) Preferential attendance at one of the practical class types is not associated with better assessment outcome.

It is clear that on the whole, there is an association between student attendance at practical classes and performance on anatomy assessment. Students who attend more appear to perform better. There are many reasons why this might be the case. In practical classes students can use a hands on approach to explore anatomical material and there is no doubt that this is affords a better learning opportunity than learning from textbooks alone. Practical classes have a role in providing a detailed view of relevant anatomy and an appreciation of relations between structures [1, 5]. Further to this, active observation and participation in cadaveric dissection helps the understanding of three-dimensional (3D) structures and reinforces knowledge provided during traditional lectures [6]. Self-exploration is an important part of this in dissection [7]. It is also possible that the extra time students devote to attending practical classes might be an important factor. This was impossible to control for in the present study. However, it is unlikely that students who do not attend a practical class spend that same amount of time studying from other resources available to them (eg. internet or textbooks), and so we speculate that the time devoted to study during these practical classes is important. Regardless of the reason, it is clear that attendance at either (or both) prosection and/or dissection classes is value-adding to assessment outcomes for anatomy students.

We could not determine whether assessment outcomes were better if students attended *only* dissection or workshop style classes, because most students in our subject attended both. We did not attempt to force students to attend one style of practical class only because we believe that both styles value-add to students’ anatomy education, and our teaching programs are designed to offer both formats to students. Thus our data can only be interpreted in the context of students who *preferentially* attended one style of practical class over the other. There is in fact only limited objective evidence for how different practical teaching methods impact on the student experience and understanding of anatomy (for a review, see [2]). Some authors argue that students who participate in prosection classes *only* out-perform students who participate in dissection classes *only* [8, 9], and others argue the opposite [10, 11]. The typical experimental design involves two groups of students that participate in either classical dissection or a different style of teaching, often based on prosected material. The two groups receive the same assessment and the results are compared to determine if statistical differences exist between the two groups. However, it appears from a systematic review of the literature [2] that groups are randomized in only half of the studies examined, and that the two groups studied are not often tightly controlled to ensure they are getting the same time devoted to the different teaching activities. In addition to this, the number of students that make up these studies is mostly small (often less than 100; [see Tables 1 and 2]). This makes it difficult to control for many variables that could influence the assessment outcomes, and is most likely why the results of these studies vary so widely. The results of our study suggest that assessment outcomes were not better for students who preferentially attended one practical class style over the other, at least in the context of our large cohort of students and within the constraints of both our practical curriculum and assessment styles. Taken together with our findings that attendance at both workshop and dissection style classes each correlate with assessment outcome, we suggest that both are important for a balanced anatomy education.

There are several limitations in this study. Whilst our findings show an association between practical class attendance and assessment outcome, there are a number of confounders that make it difficult to prove increased practical class attendance is the cause of better assessment outcome. In the current study, we assumed that the level of engagement of students studying with alternative resources, including online lecture recordings, was equal across all students. However, we were unable to derive actual lecture attendance records and/or level of engagement of individual students with other resources. Thus it is possible that students performed better on assessments because of student-level engagement and not because of the nature of the workshops/dissections. In addition, this study was not designed as a randomized trial nor did we make any effort to standardize the allocation or to compare other characteristics of high practical attenders versus low practical attenders. Finally, we could not determine whether assessment outcomes were better if students attended *only* dissection or workshop style classes, because most students in our subject attended both.

## Conclusions

Our findings show that greater student attendance at practical classes is associated with better performance on anatomy assessments, but preferential attendance at workshop or dissection style classes does not necessarily lead to better assessment outcomes.
